# Adverse events associated with acupuncture: three multicentre randomized controlled trials of 1968 cases in China

**DOI:** 10.1186/1745-6215-12-87

**Published:** 2011-03-24

**Authors:** Ling Zhao, Fu-wen Zhang, Ying Li, Xi Wu, Hui Zheng, Lin-hao Cheng, Fan-rong Liang

**Affiliations:** 1 College of Acupuncture and Massage, Chengdu University of TCM, Chengdu, Sichuan 610075, China; 2 College of Clinical Medicine, Chengdu University of TCM, Chengdu, Sichuan 610075, China; 3 College of Foreign Languages, Chengdu University of TCM, Chengdu, Sichuan 610075, China

## Abstract

**Background:**

In order to evaluate the safety of acupuncture in China objectively, we investigated the adverse events associated with acupuncture based on three multicentre randomized controlled trials (RCTs) to assess the safety of acupuncture, identifying the common types of acupuncture adverse events, and analysing the related risk factors for their occurrence.

**Methods:**

This observational study included patients who received acupuncture from three multicentre RCTs respectively for migraine, functional dyspepsia and Bell's palsy. The 1968 patients and their acupuncturists documented adverse events associated with acupuncture after treatment. We collected data about adverse events due to acupuncture treatment from their case report forms. We analysed the incidence and details of the adverse effects, and studied the risk factors for acupuncture adverse events with non-conditional logistic regression analysis.

**Results:**

Among the 1968 patients, 74 patients (3.76%) suffered at least one adverse event throughout the treatment period. We did not observe the occurrence of serious adverse events. 73 patients with adverse events recovered within 2 weeks through effective treatment such as physiotherapy or self-treatment. A total of 3 patients withdrew because of adverse events. There were 9 types of adverse events related to acupuncture, including subcutaneous haematoma, bleeding, skin bruising and needle site pain. Subcutaneous haematoma and haemorrhage in the needling points were the most common adverse events. Age and gender were related to the occurrence of acupuncture adverse events. The older the patients were, the higher the risk of adverse events was. In addition, male patients had slightly higher risk of an adverse event than female patients.

**Conclusions:**

Acupuncture is a safe therapy with low risk of adverse events in clinical practice. The risk factors for adverse events (AEs) were related to the patients' gender and age and the local anatomical structure of the acupoints. AEs could be reduced and mitigated by improving the medical environment, ensuring a high technical level of the acupuncture practitioners and establishing a good relationship of mutual trust between doctor and patient.

**Trial Registration:**

ClinicalTrials.gov: NCT00599586, NCT00599677, NCT00608660

## Background

Acupuncture has been practiced in China for thousands of years as part of the Traditional Chinese Medicine (TCM). More recently, it has gradually won acceptance in western countries as an alternative or complementary treatment for various conditions, including chronic pain syndrome [1-5]. Because of its widespread use, the safety of acupuncture is of increasing importance. Most side-effects encountered in acupuncture practice are mild, but life-threatening adverse events such as organ injury have been reported [6]. In the past few years, AEs associated with acupuncture treatment in foreign countries have been published in the form of systematic reviews or surveys [7-10], but acupuncture-related AEs that occurred in China have not received attention as many Chinese practicing acupuncturists do not regularly monitor and report AEs. In this paper we report AEs due to acupuncture in 1968 cases of patients and analyse the risk factors for their occurrence, based on three multicentre RCTs of acupuncture, to provide an objective evaluation of acupuncture safety in China. This study is expected to be useful for acupuncture practitioners as well as educators and health care decision-making departments.

## Methods

### Patients

1968 outpatients came from three multicentre RCTs in China. These three RCTs are "An RCT to treat migraine with acupuncture" (MI-RCT), "An RCT to treat functional dyspepsia with acupuncture" (FD-RCT), and "An RCT of acupuncture and moxibustion to treat Bell's palsy according to different stages" (BP-RCT).

Three RCTs were registered respectively (NCT00599586, NCT00599677, and NCT00608660) at the U.S. clinical trial registry (http://clinicaltrials.gov/). According to the criteria of the International Classification of Headache Society, patients who met the diagnosis of migraine with or without aura were included in the MI-RCT study. In the FD-RCT study, patients diagnosed with meal-induced dyspeptic symptoms and epigastric pain according to the Rome Ⅲ Diagnostic Criteria for functional Gastrointestinal Disorders. Patients meeting the diagnosis of Bell's palsy according to Chinese Medicine and Western Medicine were enrolled in the BP-RCT. The three RCTs were completed in 16 hospitals located in five provinces (Sichuan Province, Hunan Province, Hubei Province, Tianjin and Shandong Province). 1968 cases of patients were monitored from December 2007 to October 2009.

### Design

Three RCTs were performed in accordance with the principles of the Declaration of Helsinki (Edinburgh 2000 version), and trial protocols were approved by the Affiliated Hospital of Chengdu University of Traditional Chinese Medicine review board and ethics committee. The National Clinical Trial Centre of Chinese Medicine, Chengdu, Good Clinical Practice (GCP) Centre in China was responsible for the central randomisation and data management. The inclusion criteria and protocols of the three RCTs have been described in detail elsewhere [11-13].

AE is defined as an unfavourable medical event that occurs during or after the treatment regardless of causal relationship [10]. Serious adverse effects (SAEs) refers to those that caused hospitalisation, extended duration of hospitalisation, disability, impaired ability to work, death or were life threatening, resulting in events such as congenital malformations in the process of the clinical trials. AE and SAE were defined *a priori *from the literature and the State Food and Drug Administration (SFDA) in China. AEs include subcutaneous haematoma, minor haemorrhage, serious pain, fainting and local infection, and SAEs include spinal cord injury, punctured organs, convulsions and pneumothorax. All the definitions were expounded in the clinical work manual of each trial.

During the clinical trial, physicians and patients were asked to evaluate AEs/SAEs associated with acupuncture and were recorded in the case report form (CRF). After each acupuncture treatment cycle, each patient was asked to complete a questionnaire (see additional file 1: Adverse Events Questionnaire for Patients) about whether they suffered excessive pain in the needle points, nausea, dizziness, aggravation of illnesses or other discomforts during the treatment, and were evaluated if there was bleeding, haematoma, bruising, acupuncture fainting, lag needle, broken needle, forgotten needle, etc. If any of the above adverse reactions happened, doctors should record them in the AEs reports (see additional file 2: Adverse Events Reports for Acupuncturist) including the type of AE, when it occurred, how long it lasted, the severity, location, any remedial actions and when they were alleviated, the relevance to acupuncture therapy and whether the patients quitted the trial. Monitors, appointed by the trial organisers, directly tracked AEs and periodically verified the integrity and authenticity of records for quality control [14].

### Interventions

**Figure 1:**
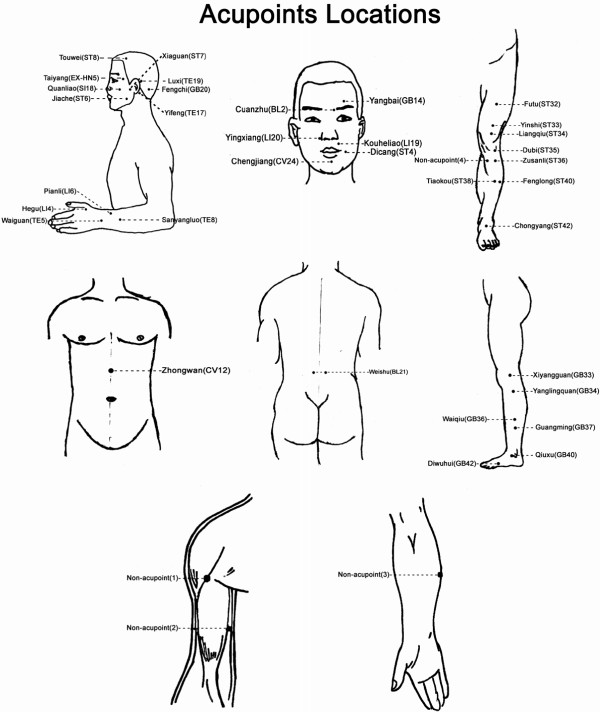
**Acupoints Locations**. Locations of acupoints used in the three randomized controlled trials.

Patients in the three RCTs were blind selected to which acupuncture treatment they received. All enrolled patients received a 4-week acupuncture treatment and a 3-month follow-up period. After randomisation, patients received 20 treatments over a period of 4 weeks, and the treatments would be administered once per day for 5 continuous days, with a 2 day rest interval. The treatment courses of the three RCTs were designed according to the acupuncture protocol used in Chinese clinical acupuncture practice. All acupuncture practitioners had undergone either at least 8 years of acupuncture training and were qualified TCM doctors or associate chief TCM doctors with more than 10 years of clinical experiences. Acupoints and manipulation procedures in MI-RCT and FD-RCT were standardised, and BP-RCT was performed using semi-standardised acupoints and standardised manipulation. Details of acupoints selected and manipulation in each trial were shown in additional file 3 (Details of acupoints selected and manipulation in each trial) and acupoints locations was shown in Figure 1. Sterile disposable one-time-use needles (Hwato Needles, Sino-foreign Joint Venture Suzhou Hua Tuo Medical Instruments Co., China) were used to achieve "de Qi" sensations. Patients in MI-RCT, FD-RCT and group 3 of BP-RCT received electro-stimulation and the other patients were stimulated manually.

### Data monitoring and Statistics

According to standard operating procedures (SOPs) for clinical research data management, clinical trial data entry and management were commissioned by the GCP centre of Chengdu using Remote Clinical Data Management Systems (RCDMS). All the results were entered twice and then checked. For inconsistent values we checked the CRFs item by item in order to ensure data accuracy. After data entry, 10 CRFs and data from the database of each trial were randomly selected and checked again to ensure consistency. After the final confirmation, all the data were imported into SPSS 13.0. After the logic programming check, the obviously incorrect data were modified. If the errors of data were from CRFs, the data manager then revised the data according to the answers from the researchers.

The SPSS 13.0 for Windows (SPSS Inc., Chicago, IL) was used for data analysis. Measurement data were indicated as mean ± SD. The acupuncture AEs related risk factors were analysed by unconditional logistic regression analysis. *P *value was noted as significant when it was less than 0.05.

## Results

### General characteristics of patients

A total of 1968 cases of patients (MI-RCT 475, FD-RCT 593, and BP-RCT 900) received acupuncture therapy in the three RCTs. The average age was 38.7 ± 14.0 years. The general characteristics of patient per trial were shown in Table 1.

**Table 1:** General Characteristics and Distribution of Patients

Characteristic		Clinical Trials			Total(%)
			
		MI-RCT	FD-RCT	BP-RCT	
*Gender*	Male	82	173	474	729 (37.0)
	Female	393	420	426	1239 (63.0)
*Marriage*	Unmarried	134	191	211	536 (27.2)
	Married	341	402	689	1432 (72.8)
*Educational Background*	Primary school	34	39	90	163 (8.3)
	Middle school	206	213	405	824 (41.9)
	University and above	235	341	405	981 (49.9)
*Acupuncture experience*	Not experienced	346	588	491	1425 (72.4)
	Have experienced	129	5	409	543 (27.6)
*BMI (kg/m*^*2*^*)*	< 18.5	53	92	24	169 (8.6)
	18.5~23.9	342	428	495	1265 (64.3)
	24.0~27.9	80	73	381	534 (27.1)
*Age (year)*	18~29	178	215	258	651 (33.1)
	30~39	104	119	191	414 (21.0)
	40~49	90	118	175	383 (19.5)
	50~59	83	105	179	367 (18.7)
	60~69	20	36	97	153 (7.8)

### AEs and sequent treatments

74 patients (3.76%) experienced AE during the observational cycle. The general information of the patients with AEs was shown in Table 2. Overall, 74 cases of AEs were recorded. The most common adverse effects were needle-site bleeding (37.84%) and subcutaneous bleeding (25.68%) (Table 3). All types of AEs observed in this study have been reported previously [7,10,15]. It was worth mentioning that the following adverse events, lag needle, broken needle and forgotten needle, were not reported, and there were no SAEs relating to organ injury or nerve injury caused by malpractice.

**Table 2:** General characteristics of patients with AEs associated with acupuncture

Characteristics		Clinical Trials			Total (%)
			
		MI-RCT	FD-RCT	BP-RCT	
*Gender*	Male	6	1	12	19 (25.68)
	Female	29	10	15	54 (72.97)
*Marriage*	Unmarried	6	1	2	9 (12.2)
	Married	30	10	25	65 (87.84)
*Educational Background*	Primary school	6	2	2	10 (13.51)
	Middle school	12	3	14	29 (39.19)
	University and above	18	6	11	35 (47.3)
*Acupuncture experience*	Not experienced	25	11	18	54 (72.97)
	Have experienced	11	0	9	20 (27.03)
*BMI (kg/m*^*2*^*)*	< 18.5	3	1	0	4 (5.4)
	18.5~23.9	30	9	18	57 (77.03)
	24.0~27.9	3	1	9	13 (17.57)
*Age (year)*	18~29	8	1	4	13 (17.57)
	30~39	7	2	9	18 (24.32)
	40~49	6	2	8	16 (21.62)
	50~59	8	4	4	16 (21.62)
	60~69	7	2	2	11 (14.86)

**Table 3:** List of AEs in acupuncture and subsequent treatments

Type of AEs		Total patients with AEs (N = 74)			Physiotherapy (N = 19)			Self-treatment (N = 37)			Others (N = 3)			No treatment (N = 15)		
		
		MI-RCT n (%)	FD-RCT n (%)	BP-RCT n(%)	MI-RCT n (%)	FD-RCT n (%)	BP-RCT n(%)	MI-RCT n (%)	FD-RCT n (%)	BP-RCT n(%)	MI-RCT n (%)	FD-RCT n (%)	BP-RCT n(%)	MI-RCT n (%)	FD-RCT n (%)	BP-RCT n(%)
*Local reactions*	Subcutaneous haematoma	6 (8.11)	3 (4.05)	23 (31.08)	6 (31.58)	3 (15.79)	3 (15.79)	0	0	9 (24.32)	0	0	0	0	0	11 (73.33)
	Minor haemorrhage in needling position	23 (31.08)	2 (2.70)	3 (4.05)	0	0	0	23 (62.16)	2 (5.41)	3 (8.11)	0	0	0	0	0	0
	Subcutaneous bruise	6 (8.11)	1 (1.35)	0	2 (10.53)	1 (5.26)	0	0	0	0	0	0	0	4 (30.77)	0	0
	Prolonged pain at the site of needling	0	1 (1.35)	0	0	1 (5.26)	0	0	0	0	0	0	0	0	0	0
*Systemic reactions*	Acupuncture fainting	0	1 (1.35)	1 (1.35)	0	0	0	0	0	0	0	1 (33.33)	1 (33.33)	0	0	0
	Abdominal distension	0	1 (1.35)	0	0	1 (5.26)	0	0	0	0	0	0	0	0	0	0
	Dizziness/vertigo	0	1 (1.35)	0	0	0	0	0	0	0	0	1 (33.33)	0	0	0	0
	Leg weakness	1(1.35)	0	0	1 (5.26)	0	0	0	0	0	0	0	0	0	0	0
	Muscle spasm	0	1 (1.35)	0	0	1 (5.26)	0	0	0	0	0	0	0	0	0	0

59 patients (79.73%) received subsequent treatment for recovery from AEs, including physiotherapy (19, 32.20%), self-treatment (37, 62.71%) and other effective methods (3, 5.08%) (Table 3). 73 patients with AEs recovered in 2 weeks after subsequent treatment. One patient with dizziness did not fully recover within 2 weeks but reached full recovery one month after the cessation of acupuncture treatment. Of these 74 patients, three patients suffered either muscle spasm or dizziness or abdominal distension and withdrew from the study. The remaining 71 patients completed the entire treatment process. 64 of these 74 AEs cases were certainly due to the acupuncture treatment, and the remaining 10 cases were probably related to the acupuncture treatment.

### Logistic regression analysis of AE risk factors related with acupuncture treatment

We analysed various potential risk factors associated with acupuncture treatment using logistic regression. We set the occurrence of AEs associated with acupuncture as a dependent variable (two categories, "1" for no AE, "2" for AE), and age (continuous variable), sex (two categories, "1" for male, "2" for female ), education (multi-categorical variables, "1" for primary school education level, "2" for high school education level, "3" for university education level and above ), history of acupuncture treatment (two categories, "1" for without acupuncture experience, "2" for with acupuncture experience) and body mass index (continuous variable) were set as covariates. All the variables were adopted into the model for analysis by using forward stepwise selection of independent variables for Wald Probability and Statistics Act for Logistic Regression analysis. Logistic regression equation was: Logit (AE) = -3.782 + 0.018 * age + (-0.539 * gender); regarding the relative risk factor (Exp (B)) value terms, with one year increase, the possibility of AEs would increase 1.018-1 = 0.018 times. From the perspective of gender, the possibility of AEs for females is 0.583-1 = -0.417 times that for males, i.e. the possibility of AEs for females is 0.417 times less than for males (Tables 4 and 5).

**Table 4:** Variables not included in the analysis of the model

			Score	df	*p*
Step 1	Variables	Gender (1)	4.055	1	0.044
		Educational Background	1.141	2	0.565
		Educational Background (1)	0.932	1	0.334
		Educational Background (2)	0.644	1	0.422
		Acupuncture experience (1)	0.966	1	0.326
		BMI	2.649	1	0.104
	Overall Statistics		7.407	5	0.192
Step 2	Variables	Educational Background	0.824	2	0.662
		Educational Background (1)	0.554	1	0.456
		Educational Background (2)	0.607	1	0.436
		Acupuncture experience (1)	1.199	1	0.274
		BMI	1.422	1	0.233
	Overall Statistics		3.382	4	0.496

**Table 5:** Logistic Regression analysis for variables included in the Model

		B	S.E.	Wald	df	*P*	Exp(B)	95% C.I. for EXP(B)	
								
								Lower	Upper
Step 1^a^	Age	0.018	0.008	4.897	1	0.027	1.018	1.002	1.034
	Constant	-3.965	0.361	120.618	1	0.000	0.019		
Step 2^b^	Age	0.018	0.008	4.719	1	0.030	1.018	1.002	1.034
	Gender(1)	-0.539	0.271	3.967	1	0.046	0.583	0.343	0.991
	Constant	-3.782	0.369	105.284	1	0.000	0.023		

Notes: a. Variable(s) entered on step 1: age; b. Variable(s) entered on step 2: gender.

## Discussion

With the increasing acceptance of acupuncture in more and more countries, governments and professional institutions, the safety of acupuncture is becoming of key concern in public discussion. Every acupuncture practitioner should objectively and factually report acupuncture AEs. According to the reports from United States, Germany, Britain, Korea, Japan et al [10,16-18], the incidence of AEs ranged from 0.671% to 11.4%, with the most common acupuncture AEs being pain, fatigue, bleeding and haematoma. In this study, in order to ensure real and objective safety evaluation of acupuncture, we used a synchronous mode of observational studies to obtain the first-hand research data. The 1968 patients and their physicians were required to complete an acupuncture AE questionnaire and acupuncture adverse reports respectively. They were supervised and spot-checks were made during the whole process to minimize omission and selection bias. Since after-effects of acupuncture are known to exist, the study period to evaluate AEs included the acupuncture treatment process and the three months after treatment.

According to the type and frequency of AEs in acupuncture treatment established by Witt [19], ecchymoma and haemorrhage in needling position were the most common AEs, with an occurrence rate of more than 10%. Our results were consistent with the Witt's. Previous literatures indicated there were 9 kinds of AEs, but the incidence and types of AEs found in our study were lower than former records. Comparing relevant literatures, we inferred that acupuncture AEs were associated with following factors.

Firstly, acupuncture AEs were related to the patient's own attributes. In this study, according to the results of logistic regression analysis, the occurrence of acupuncture AEs did not have significant correlation to BMI, educational background and acupuncture treatment experience of patients, but was correlated to age and sex. The older the patient, the higher was the risk of AE, and male patients had slightly higher risk of AE than female patients. The analysis of risk factors for acupuncture AEs has only rarely been studied in previous literatures, usually only case reports have been reported. We have done these preliminary studies but still need to collect more demographic information and details to further this investigation.

Secondly, the types of acupuncture AEs were connected to the location and anatomical structure of the acupoints. In our study, the most common AEs of acupuncture were ecchymoma and bleeding in the needling position. From the occurrence percentage of the two types of AEs, subcutaneous haematoma accounted for 23/32 (71.88%) in acupuncture treatment for Bell's palsy, and bleeding in the needling position accounted for 23/28 (82.14%) in the therapy of migraine. The treatment plan of peripheral facial paralysis involved Yangbai (GB14), Dicang (ST4), Jiache (ST6), Xiaguan (ST7), Taiyang (EX-HN5), Quanliao (SI18) and other facial acupoints. As facial skin is thin and rich in blood vessels and superficial fascia are composed of loose connective tissue with plentiful vessels, it is easy to cause vessel damage or latent subcutaneous bleeding during needle manipulation. In the treatment of migraine, we chose Fengchi (GB20), Luxi (TE19), and Touwei (ST8) which are located on cranial region of head. As the cranial skin and superficial fascia have abundant blood circulation, and fibrous connective tissue under the superficial fascia and the vessel wall are closely linked, scalp bleeding is common after acupuncture and it is difficult to self-terminate as pressure needs to be applied to stop the bleeding. Thus, mastering the anatomical characteristics of acupoints accurately could not only prevent the occurrence of AEs but also contribute to treating adverse reactions actively and minimize the harm to patients.

Thirdly, AEs were related to the medical environment. The 1968 patients of three RCTs were from 16 hospitals in five provinces. These medical institutions are all public medical institutions and are top hospitals in China. All patients received acupuncture treatment using disposable needles in order to avoid cross-infection such as needle site infection, the emergence of sepsis and hepatitis, which can be caused by defective needle disinfection [20].

Fourthly, we inferred that the incidence of acupuncture AEs and acupuncture practitioner's experience and skills are probably and inversely related. In public hospitals of China, acupuncture physician should have obtained qualifications for a doctor's license before treating patients. This means that an acupuncture physician has received at least 4 years university education and at least 1 year clinical experience, not including the period of internship. The education background and working experience of all acupuncture practitioners in these three RCTs were very similar, they had undergone at least 8 years of acupuncture training and were qualified TCM doctors. Additionally, before the beginning of these clinical trials, all the acupuncture practitioners had received SOPs training courses of acupuncture operation plan. Only the acupuncturists who finished the training course and passed the qualification examination were able to take part in the clinical trial. During the process of acupuncture treatment, every trial unit was required to have at least two acupuncturists with more than 10 years of clinical experience, as technical advisors to provide guidance to young practitioners. We made the above inference by comparing the education background and qualifications of acupuncture physician reported in most of the foreign countries [5,21,22] (e.g. Modlock et al. described that midwives were trained in acupuncture according to the guidelines and performed acupuncture treatments five to six times a week) with those in our study. The level of training may be one of the reasons for the difference in frequency and types of acupuncture AEs between China and foreign countries.

Lastly, we considered that the mutual trust between doctors and patients could reduce the occurrence of AEs. The level of communication between doctors and patients during the process of clinical trials could be important so that all the patients were able to follow the doctor's instruction, for example, do not receive acupuncture treatment in a fasting state or satiated state. To some degree, such measures could help reduce acupuncture syncope and aggravation of the disease caused by improper care.

The limitation of this study was it involved only migraine, functional dyspepsia, and Bell's palsy, without relating to other appropriate disease in acupuncture, and two-thirds data about acupuncture safety in this manuscript were from the trials for efficacy study.

## Conclusions

Our study confirmed that acupuncture is a safe therapy. The risk factors for AEs were related to the patient's gender and age and the local anatomical structure at the acupoints. AEs could be reduced and mitigated by improving the medical environment, promoting the technical level of the acupuncture practitioners, and establishing a good relationship of mutual trust between doctor and patient. As acupuncture practitioners, we should be alert to the occurrence of acupuncture AEs. Once AEs taking place, we ought to handle and report them with scientific, rigorous and serious attitudes. Acupuncture training institutions, educators and medical decision-making departments should attach great importance to the provision of professional standards and skill training for acupuncture practitioners in order to minimize the risk of AEs.

## List of abbreviations

RCT: Randomized controlled trial; AE: Adverse event; TCM: Traditional Chinese Medicine; MI-RCT: An RCT to treat migraine with acupuncture; FD: Functional dyspepsia; FD-RCT: An RCT to treat functional dyspepsia with acupuncture; BP-RCT: An RCT of acupuncture and moxibustion to treat Bell's palsy according to different stages; GCP: Good Clinical Practice; SAE, Serious adverse effect; SFDA: the State Food and Drug Administration; CRF: Case report form; SOP: Standard operating procedures; RCDMS: Remote Clinical Data Management Systems

## Competing interests

The authors declare that they have no competing interests.

## Authors' contributions

LZ drafted the manuscript. FWZ performed the data analysis. All authors contributed to the further writing of the manuscript as well as read and approved the final manuscript.

## Supplementary Material

Additional file 1 Adverse Events Questionnaire for Patients Click here for file

Additional file 2 Adverse Events Reports for Acupuncturist Click here for file

Additional file 3 Details of acupoints selected and manipulation in each trial Click here for file
