# Supplementary material for: Adverse events associated with acupuncture: three multicentre randomized controlled trials of 1968 cases in China
Source: Trials. 2011 Mar 24;12:87. doi: 10.1186/1745-6215-12-87 (PMC3072923; doi:10.1186/1745-6215-12-87)
Supplement: Additional file 1 — Adverse Events Questionnaire for Patients [file 1745-6215-12-87-S1.DOC]

## Additional file 1 Adverse Events Questionnaire for Patients

Have you ever experienced any adverse symptoms associated with acupuncture? If yes, please mark "√" in the following table.

|  | Frequency | | | | | | | |
| --- | --- | --- | --- | --- | --- | --- | --- | --- |
|  | 1 | 2 | 3 | 4 | 5 | 6 | 7 | 8 |
| Excessive pain in needle points |  |  |  |  |  |  |  |  |
| Haematoma |  |  |  |  |  |  |  |  |
| Bruise |  |  |  |  |  |  |  |  |
| Infection |  |  |  |  |  |  |  |  |
| Dizziness/vertigo |  |  |  |  |  |  |  |  |
| Nausea |  |  |  |  |  |  |  |  |
| Aggravation of illnesses |  |  |  |  |  |  |  |  |
| Acupuncture fainting |  |  |  |  |  |  |  |  |
| Organ injury/nerve injury |  |  |  |  |  |  |  |  |
| Lag needle |  |  |  |  |  |  |  |  |
| Broken needle |  |  |  |  |  |  |  |  |
| Forgotten needle |  |  |  |  |  |  |  |  |
| Other discomforts (Please specify adverse effects and frequency of each): | | | | | | | | |
|  | | | | | | | | |
