# Supplementary material for: Adverse events associated with acupuncture: three multicentre randomized controlled trials of 1968 cases in China
Source: Trials. 2011 Mar 24;12:87. doi: 10.1186/1745-6215-12-87 (PMC3072923; doi:10.1186/1745-6215-12-87)
Supplement: Additional file 2 — Adverse Events Reports for Acupuncturist [file 1745-6215-12-87-S2.DOC]

## Additional file 2 Adverse Events Reports for Acupuncturist

| **Adverse Events** | **Occurrence time** | **Interventions** | **Ending time** | **Alleviative situation** | **Correlation with acupuncture** | **Withdrew from trial** |
| --- | --- | --- | --- | --- | --- | --- |
|  |  |  |  | □ Recover  □Recovery but leave sequela  □ Dead  □ Unknown  □ Not return | □ Certainly  □ Probably/likely  □ Possibly  □ Unlikely  □ Unassessable | □ Yes  □ No |
|  |  |  |  | □ Recover  □Recovery but leave sequela  □ Dead  □ Unknown  □ Not return | □ Certainly  □ Probably/likely  □ Possibly  □ Unlikely  □ Unassessable | □ Yes  □ No |
