# Supplementary material for: Adverse events associated with acupuncture: three multicentre randomized controlled trials of 1968 cases in China
Source: Trials. 2011 Mar 24;12:87. doi: 10.1186/1745-6215-12-87 (PMC3072923; doi:10.1186/1745-6215-12-87)
Supplement: Additional file 3 — Details of acupoints selected and manipulation in each trial [file 1745-6215-12-87-S3.DOC]

## Additional file 3 Details of acupoints selected and manipulation in each trial

| Trials | Groups | Acupoints | Manipulations |
| --- | --- | --- | --- |
| **MI-RCT** | Group 1 | 1. Waiguan (TE5) 2. Yanglingquan (GB34) 3. Qiuxu (GB40) 4. Fengchi (GB20) | 1. is punctured perpendicularly 0.5–1 cun 2. is punctured perpendicularly 1–1.5 cun 3. is punctured perpendicularly 0.5–0.8 cun 4. is punctured obliquely 0.8–1.2 cun, the tip of needle towards the tip of the nose |
|  | Group 2 | 1. Luxi (TE19) 2. Sanyangluo (TE8) 3. Xiyangguan (GB33) 4. Diwuhui (GB42) | 1. is punctured transversely 0.3–0.5 cun 2. is punctured perpendicularly 0.5–1cun 3. is punctured perpendicularly 1–1.5 cun 4. is punctured perpendicularly 0.5–0.8 cun |
|  | Group 3 | 1. Touwei (ST8) 2. Pianli (LI6) 3. Zusanli (ST36) 4. Chongyang(ST42) | 1. is punctured transversely 0.5–1.0 cun 2. is punctured perpendicularly 0.3–0.5 cun 3. is punctured perpendicularly 1–2 cun 4. is punctured perpendicularly 0.3–0.5 cun, avoid needling the artery |
|  | Group 4 | 1. Non-acupoint 1 2. Non-acupoint 2 3. Non-acupoint 3 4. Non-acupoint 4 | 1. is punctured perpendicularly 0.5–1 cun 2. is punctured perpendicularly 0.5–1 cun 3. is punctured perpendicularly 0.5–1 cun 4. is punctured perpendicularly 0.5–1 cun |
| **FD-RCT** | Group 1 | 1. Chongyang(ST42) 2. Fenglong(ST40) 3. Zusanli(ST36) 4. Liangqiu(ST34) | 1. is punctured perpendicularly 0.3–0.5 cun, avoid needling the artery 2. is punctured perpendicularly 1–1.5 cun 3. is punctured perpendicularly1–2 cun 4. is punctured perpendicularly 1–1.2 cun |
|  | Group 2 | 1. Tiaokou(ST38) 2. Dubi(ST35) 3. Yinshi(ST33) 4. Futu(ST32) | 1. is punctured perpendicularly 1–1.5 cun 2. is punctured perpendicularly 0.5–1 cun 3. is punctured perpendicularly 1–1.5 cun 4. is punctured perpendicularly 1–2 cun |
|  | Group 3 | (1) Weishu(BL21)  (2) Zhongwan(CV12) | (1) is punctured obliquely along the spine for 0.5–0.8 cun  (2) is punctured perpendicularly 1–1.5 cun |
|  | Group 4 | 1. Qiuxu(GB40) 2. Guangming(GB37) 3. Yanglingquan(GB34) 4. Waiqiu(GB36) | 1. is punctured perpendicularly 0.5–0.8 cun 2. is punctured perpendicularly 0.5–0.8 cun 3. is punctured perpendicularly 1–1.5 cun 4. is punctured perpendicularly 0.5–0.8 cun |
|  | Group 5 | The acupoints and manipulation are the same as the group 4 in the MI-RCT.. | |
| **BP-RCT** | Group 1 | **Main acupoints:**   1. Yangbai (GB14) 2. Dicang (ST4) 3. Jiache (ST6) 4. Xiaguan (ST7) 5. Taiyang (EX-HN5) 6. Quanliao (SI18) 7. Yifeng(TE17) 8. Hegu (LI4)   **Additional acupoints:**  (1)Yingxiang (LI20)   1. Kouheliao (LI19) 2. Cuanzhu (BL2) 3. Chengjiang (CV24)   The main acupoints could be used at each stage, and the additional acupoints could be selected individually, according to patient symptoms. | Main acupoints (1) to (7) are punctured unilaterally. At acute and resting stages, Hegu(LI4) is used at uninjured side, and other acupoints are used at paralyzed side. At restoration stage, Hegu (LI4) and Zusanli (ST36) are used for both sides, while other acupoints are only used at paralyzed side. At acute stage, shallow puncturing is used at facial acupoints and routine puncturing is used at other acupoints. Yifeng (TE17), Hegu (LI4) are punctured 0.5-1.0 cun, the others are punctured 0.1-0.3 cun. At resting stage, penetrative needling is used from Dicang (ST4) to Jiache (ST6) and from Taiyang (EX-HN5) to Quanliao (SI18) 2-3cun, routine puncturing is used at other acupoints. At restoration stage, the manipulation is the same as the resting stage, while Hegu (LI4) and Zusanli (ST36) are used at both sides. |
|  | Group 2 |  | The acupoints and manipulation of each stage are the same as the group 1. Meanwhile, mild moxibustion with moxa stick is applied at each main acupoints for 5 min after finishing needling. |
|  | Group 3 |  | The acupoints and manipulation of the acute stage are the same as the group1. At resting stage, electro-acupuncture is used at Dicang (ST4) and Xiaguan (ST7), Taiyang (EX-HN5) and Yangbai (GB14) by SDZ-II (made in Suzhou, China). The stimulation frequency is 50-60 times a minute. The stimulation intensity is varied till the patients feel comfortable. And routine puncturing is used at other acupoints. At restoration stage, the manipulation is the same as the resting stage, while Hegu (LI4) and Zusanli (ST36) are used at both sides. |
|  | Group 4 |  | The acupoints and manipulation of each stage are the same as the group 1. Meanwhile acupuncture along yangming musculature is used at resting and restoration stages. Four or five needles are punctured and ranked in one line along yangming musculature, and eight or ten needles are ranked in two lines totally. Hegu (LI4) and Zusanli (ST36) are used bilaterally at restoration stage. |
|  | Group 5 |  | The main and secondary acupoints are the same as those of the group 1. Hegu (LI4) is used at uninjured side and others at paralyzed side. The routine puncturing is used at each acupoint regardless of path-stage affiliation. At the acute stage retaining the needles for 20 min without manipulation is required, while at the resting and restoration stages are required to retain the needles for 30 min, manipulating the needles once at each 15 min interval. |
